# Coexistence of Low Vitamin D and High Fibroblast Growth Factor-23 Plasma Levels Predicts an Adverse Outcome in Patients with Coronary Artery Disease

**DOI:** 10.1371/journal.pone.0095402

**Published:** 2014-04-18

**Authors:** José Tuñón, Carmen Cristóbal, Nieves Tarín, Álvaro Aceña, María Luisa González-Casaus, Ana Huelmos, Joaquín Alonso, Óscar Lorenzo, Emilio González-Parra, Ignacio Mahíllo-Fernández, Ana María Pello, Rocío Carda, Jerónimo Farré, Fernando Rodríguez-Artalejo, Lorenzo López-Bescós, Jesús Egido

**Affiliations:** 1 Department of Cardiology, IIS-Fundación Jiménez Díaz, Madrid, Spain; 2 Laboratory of Vascular Pathology, IIS-Fundación Jiménez Díaz, Madrid, Spain; 3 Autónoma University, Madrid, Spain; 4 Department of Cardiology, Hospital de Fuenlabrada, Fuenlabrada, Spain; 5 Rey Juan Carlos University, Alcorcón, Spain; 6 Department of Cardiology, Hospital Universitario de Móstoles, Madrid, Spain; 7 Laboratory of Nephrology and Mineral Metabolism, Hospital Gómez-Ulla, Madrid, Spain; 8 Department of Cardiology, Hospital Universitario Fundación Alcorcón, Madrid, Spain; 9 Department of Nephrology, IIS-Fundación Jiménez Díaz, Madrid, Spain; 10 Department of Epidemiology, IIS-Fundación Jiménez Díaz, Madrid, Spain; 11 Department of Preventive Medicine and Public Health, School of Medicine, Universidad Autónoma de Madrid/IdiPaz and CIBERESP, Madrid, Spain; 12 CIBERDEM, Madrid, Spain; University of Bologna, Italy

## Abstract

**Objective:**

Vitamin D and fibroblast growth factor-23 (FGF-23) are related with cardiovascular disorders. We have investigated the relationship of calcidiol (vitamin D metabolite) and FGF-23 plasma levels with the incidence of adverse outcomes in patients with coronary artery disease.

**Methods:**

Prospective follow-up study of 704 outpatients, attending the departments of Cardiology of four hospitals in Spain, 6–12 months after an acute coronary event. Baseline calcidiol, FGF-23, parathormone, and phosphate plasma levels were assessed. The outcome was the development of acute ischemic events (any acute coronary syndrome, stroke, or transient ischemic attack), heart failure, or death. Cox regression adjusted for the main confounders was performed.

**Results:**

Calcidiol levels showed a moderate-severe decrease in 57.3% of cases. Parathormone, FGF-23, and phosphate levels were increased in 30.0%, 11.5% and 0.9% of patients, respectively. Only 22.4% of patients had glomerular filtration rate<60 ml/min1.73 m^2^. After a mean follow-up was 2.15±0.99 years, 77 patients developed the outcome. Calcidiol (hazard ratio [HR] = 0.67; 95% confidence interval [CI] = 0.48–0.94; p = 0.021) and FGF-23 (HR = 1.13; 95% CI = 1.04–1.23; p = 0.005) plasma levels predicted independently the outcome. There was a significant interaction between calcidiol and FGF-23 levels (p = 0.025). When the population was divided according to FGF-23 levels, calcidiol still predicted the outcome independently in patients with FGF-23 levels higher than the median (HR = 0.50; 95% CI = 0.31–0.80; p = 0.003) but not in those with FGF-23 levels below this value (HR = 1.03; 95% CI = 0.62–1.71; p = 0.904).

**Conclusions:**

Abnormalities in mineral metabolism are frequent in patients with stable coronary artery disease. In this population, low calcidiol plasma levels predict an adverse prognosis in the presence of high FGF-23 levels.

## Background

Renal disease is associated with increased cardiovascular risk. When mild decrements in glomerular filtration rate appear, phosphate elimination decreases. High phosphate serum levels have been associated with increased mortality and cardiovascular events even in the absence of renal disease [1]. Compensatory responses to maintain phosphate homeostasis include an increase in fibroblast growth-23 (FGF-23) and a subsequent rise in parathormone levels [2]. FGF-23 promotes phosphaturia and diminishes vitamin D plasma levels, downregulating its synthesis and enhancing its degradation [2].

Adaptive mechanisms appear before serum phosphate levels are increased. These mechanisms have also been related to vascular damage [3], [4]. High FGF-23 plasma levels are independently associated with endothelial dysfunction, arterial stiffness, left ventricular hypertrophy, progression of renal disease, incidence of mortality and cardiovascular events [3], [4]. Vitamin D deficiency has been associated with hypertension, coronary artery disease (CAD) and stroke [5], [6]. In addition, vitamin D attenuates left ventricular hypertrophy [7], [8]. However, no information is available regarding the possible influence of the relationship between these molecules on the prognosis of patients with CAD.

We have studied 704 patients with CAD in order to assess whether plasma levels of calcidiol (a vitamin D metabolite) and FGF-23 are related to the incidence of adverse outcomes, and the potential influence of their relationship on the prognosis.

## Methods

### Patients

The BACS & BAMI (Biomarkers in Acute Coronary Syndrome & Biomarkers in Acute Myocardial Infarction) studies included patients admitted to four hospitals in Madrid with either non-ST elevation acute coronary syndrome (NSTEACS) or ST-elevation myocardial infarction (STEMI). NSTEACS was defined as rest angina lasting more than 20 minutes in the previous 24 hours, or new-onset class III-IV angina, along with transient ST depression or T wave inversion in the electrocardiogram considered diagnostic by the attending cardiologist and/or troponin elevation. STEMI was defined as symptoms compatible with angina lasting more than 20 minutes and ST elevation in two adjacent leads in the electrocardiogram without response to nitroglycerin, and troponin elevation. Exclusion criteria were: age over 85 years, coexistence of other significant cardiac disorders except left ventricular hypertrophy secondary to hypertension, coexistence of any illness or toxic habits that could limit patient survival, impossibility to perform revascularization when indicated, and subjects in whom follow-up was not possible. In order to avoid variability of findings due to an excessive heterogeneity in the intervals between the acute event and blood extraction, the investigators agreed to exclude patients that were not clinically stable the sixth day after the index event.

In addition to plasma extraction at discharge, a second plasma sample was obtained between six and twelve months later, when the patients were stable. The present paper is a sub-study of BACS & BAMI studies, and reports data from the clinical and analytic findings obtained at the moment of this extraction, relating them to subsequent follow-up.

Between July 2006 and April 2010, 1,898 patients were discharged from the study hospitals with a diagnosis of NSTEACS or STEMI. Eight hundred thirty-eight patients were included in the study. The remaining patients were not included due to the following: age over 85 years (17.3%), presence of disorders or toxic habits limiting survival (29.0%), impossibility to perform cardiac revascularization (14.5%), coexistence of other significant cardiopathy (6.8%), impossibility to perform follow-up (12.0%), clinical instability beyond the sixth day after the index event (9.1%), refusal to participate in the study (2.0%), and impossibility of the investigators to include them (9.3%). Of the 838 patients included in the acute phase, 7 died before the second visit, and 709 had adequate plasma samples withdrawn six to twelve months after being discharged. These patients were included in the present sub-study. Plasma extraction and baseline visits took place between January 2007 and February 2011. Last follow-up visits were carried out on May 2012.

### Ethics Statement

The research protocol conforms to the ethical guidelines of the 1975 Declaration of Helsinky as reflected in a priori approval by the human research committees of the institutions participating in this study: Fundación Jiménez Díaz, Hospital Fundación Alcorcón, Hospital de Fuenlabrada y Hospital Universitario de Móstoles. All patients signed informed consent documents.

### Study Design

At baseline, clinical variables were recorded and twelve-hour fasting venous blood samples were withdrawn and collected in EDTA. Blood samples were centrifuged at 2,500 g for 10 minutes and plasma was stored at −80°C. Patients were seen every year at their hospital. At the end of follow-up (maximum 4.6 years) the medical records were reviewed and patient status was confirmed by telephone contact.

The outcome was the combination of acute ischemic events (NSTEACS, STEMI, stroke and transient ischemic attack) plus heart failure and all-cause mortality. NSTEACS and STEMI were defined as explained above. A past myocardial infarction was diagnosed in the presence of new pathological Q waves in the electrocardiogram along with a concordant new myocardial scar identified either by echocardiography or nuclear magnetic resonance imaging. Stroke was defined as rapid onset of a neurologic deficit attributable to a focal vascular cause lasting more than 24 hours or supported by new cerebral ischemic lesions at imaging studies. A transient ischemic attack was defined as a transient stroke with signs and symptoms resolving before 24 hours without cerebral acute ischemic lesions at imaging techniques. Heart failure was a clinical diagnosis made in accordance to practice guidelines [9]. Events were adjudicated by at least two investigators of the study, along with a neurologist for cerebrovascular events. Although all events were recorded for each case, patients were excluded from the Cox regression analysis after the first event. Then, although the total number of events is described, patients that had more than one event were computed only once for these analyses.

### Biomarker and Analytical Studies

Plasma determinations were performed at the laboratory of Nephrology at the Gómez-Ulla hospital and at the Biochemistry Laboratory at Fundación Jiménez Díaz. The investigators who performed the laboratory studies were unaware of clinical data. Plasma calcidiol levels were quantified by chemiluminescent immunoassay (CLIA) on the LIAISON XL analyzer (LIAISON 25OH-Vitamin D total Assay DiaSorin, Saluggia, Italy), FGF-23 was measured by an enzyme-linked immunosorbent assay which recognizes epitopes within the carboxyl-terminal portion of FGF-23 (Human FGF-23, C-Term, Immutopics Inc, San Clemente, CA), intact parathormone was analyzed by a second-generation automated chemiluminescent method (Elecsys 2010 platform, Roche Diagnostics, Mannheim, Germany), phosphate was determined by an enzymatic method (Integra 400 analyzer, Roche Diagnostics, Mannheim, Germany), and high-sensitivity C-reactive protein was assessed by latex-enhanced immunoturbidimetry (ADVIA 2400 Chemistry System, Siemens, Germany). Lipids, glucose and creatinine determinations were performed by standard methods (ADVIA 2400 Chemistry System, Siemens, Germany).

### Statistical Analysis

Quantitative data following a normal distribution are presented as mean±standard deviation (SD), those not normally distributed are displayed as median (interquartile range), and qualitative variables are presented as percentages. Also, correlations between quantitative variables were assessed with the Spearman’s rho coefficient (r).

Given the existence of four clinically relevant categories [10], calcidiol levels were assessed using these categories instead of studying it as a continuous variable: 0.00–10.00 ng/ml (severe deficiency), 10.01–20.00 ng/ml (moderate deficiency), 20.01–30.00 ng/ml (insufficiency/suboptimal levels), and higher than 30.00 ng/ml (sufficiency). In univariate analyses, the association of categories of calcidiol and FGF-23 with the adverse outcome was summarized by Kaplan-Meier curves. Differences in baseline data of patients meeting the primary outcome as compared to those remaining stable were assessed using χ^2^ or Fisher exact test for qualitative data. For quantitative variables, a Student’s t-test was performed for those following a normal distribution, and the Mann-Whitney test was used in those not normally distributed. All variables that showed significant differences in these tests (Table 1) were entered into a multivariate Cox model with stepwise forward selection of variables. In this model, the interaction between calcidiol and FGF-23 was assessed with an interaction term defined as the product of those variables dichotomized using the sample median; statistical significance was assessed with the Wald test. Given that “p” value for interaction was 0.025, analyses were repeated in two strata defined by the median of FGF-23.

**Table 1:** Baseline characteristics of patients.

	Patients Without Events (N = 627)	Patients with Events (N = 77)	P value
**Age (yr)**	59.0 (51.0–71.0)	72.0 (61.5–77.0)	**<0.001**
**Male sex (%)**	76.7	63.6	**0.017**
**Caucasian (%)**	97.0	97.4	1.000
**Body-mass index (Kg/m2)**	28.4 (25.7–30.8)	28.6 (25.7–34.1)	0.134
**Diabetes (%)**	22.0	33.8	**0.031**
**Present smoker (%)**	6.7	5.2	0.808
**Hypertension (%)**	62.4	88.3	**<0.001**
**Peripheral artery disease (%)**	3.3	7.8	0.105
**Cerebrovascular events (%)**	3.0	6.5	0.170
**Previous CABG (%)**	7.3	16.9	**0.008**
**Previous PCI (%)**	78.1	77.9	1.000
**Ejection fraction<40% (%)**	11.6	13.0	**0.016**
**Past or present Atrial fibrillation (%)**	3.3	16.9	**<0.001**
**Aspirin (%)**	92.7	85.7	**0.045**
**Clopidogrel (%)**	68.6	61.0	0.197
**Acenocumarol (%)**	4.6	15.6	**0.001**
**Statins (%)**	88.7	79.2	**0.026**
**Oral antidiabetic drugs (%)**	17.4	20.8	0.434
**Insulin (%)**	5.4	14.3	**0.006**
**ACEI/ARB (%)**	71.0	64.9	0.291
**Aldosterone receptor blockers**	5.1	11.7	**0.034**
**Betablockers (%)**	77.2	70.1	0.200
**Verapamil (%)**	0.5	0.0	1.000
**Diltiazem (%)**	2.7	7.8	**0.031**
**Dihydropyridines (%)**	15.9	22.1	0.193
**Diuretics (%)**	17.7	32.5	**0.003**
**Digoxin (%)**	0.3	1.3	0.294
**DATA FROM LAST ACS**			
** STEMI/NSTEACS (%)**	39.6/60.4	31.2/68.8	0.173
** Number of vessels diseased**	1.35±0.80	1.57±0.83	0.022
** Left main disease (%)**	3.8	5.2	0.804
** Revascularization (%)**	80.5	72.7	0.136
** Type of revascularization**			0.331
** CABG (%)**	4.9	5.2	
** Drug-eluting stent (%)**	47.6	36.4	
** Conventional stent (%)**	25.8	29.9	
** Balloon angioplasty (%)**	2.1	1.3	
** No revascularization (%)**	19.5	27.3	
** Complete revascularization (%)**	67.3	49.1	**0.007**
**ANALYTICAL DATA**			
** LDL cholesterol (mg/dl)**	82.7±24.2	86.7±35.2	0.335
** HDL cholesterol (mg/dl)**	43.7±10.7	45.2±12.2	0.250
** Non-HDL colesterol (mg/dl)**	108.3±29.7	114.0±39.6	0.224
** Triglycerides (mg/dl)**	130.4±84.7	136.8±66.7	0.522
** Glycemia (mg/dl)**	109.0±33.3	110.2±46.9	0.786
**GFR (MDRD) (ml/min/1.73** **m^2^)**	73.4 (61.9–84.1)	64.1 (48.2–79.2)	**<0.001**
** HS C-reactive protein (mg/L)**	4.41±9.73	5.20±9.11	0.499
** Calcidiol (ng/ml)**	19.0 (14.0–24.7)	16.5 (10.6–23.4)	**0.003**
** Calcidiol range**			**0.004**
**≤10 ng/ml**	9.5	22.1	
**10.01–20.0 ng/ml**	46.3	46.8	
**20.01–30 ng/ml**	33.1	26.0	
**>30.0 ng/ml**	11.1	5.2	
** FGF-23 (RU/ml)**	69.3 (53.9–93.1)	85.0 (61.9–118.2)	**0.003**
** Parathormone (pg/ml)**	59.4 (44.4–75.9)	65.1 (48.1–93.0)	**0.025**
** Phosphate (mg/dl)**	3.23±1.38	3.35±0.61	0.489

Categorical variables are presented as percentages, quantitative variables with normal distribution as mean±SD and those not normally distributed as median (interquartile range).

ACEI: Angiotensin Converting Enzyme Inhibitors; ACS: Acute coronary syndrome; ARB: Angiotensin receptor blockers; CABG: Coronary Artery by-pass graft; FGF-23: Fibroblast Growth Factor-23; GFR: Glomerular Filtration Rate; HDL: High-density lipoprotein; HS: High-Sensitivity; LDL: Low-density lipoprotein; MDRD: Modification of diet in renal disease; NSTEACS: Non-ST Elevation Acute Coronary Syndrome; PCI: Percutaneous coronary Intervention; STEMI: ST-Elevation Myocardial Infarction.

Analyses were performed with SPSS 19.0 (SPSS Inc., New York), and were considered significant when “p” was lower than 0.05 (two-tailed).

## Results

### Baseline Characteristics of the Patients

From the 709 patients included, 5 were lost to follow-up, leaving a total of 704 for analysis. Age was 61.4±12.3 years. Estimated glomerular filtration rate was 72.0±18.0 ml/min1.73 m^2^ (Modification of Diet in Renal Disease Method) and 22.4% of patients had this value below 60 ml/min 1.73 m^2^. Time from last acute coronary event was 7.5±3.0 months. Four patients (0.6%) were taking vitamin D supplements.

Calcidiol levels were up to 10.0 ng/ml in 10.9% of patients, 10.01–20.00 ng/ml in 46.4%, 20.01–30.0 ng/ml in 32.3% and higher than 30 ng/dl in 10.4%. Parathormone levels were 59.7 (45.5–77.5) pg/ml, and 30% of the patients displayed increased levels (higher than 74 pg/ml). Phosphate levels were 3.2 (2.8–3.5) mg/dl, with 0.9% of cases with values above 4.5 mg/dl. FGF-23 levels were 69.9 (54.5–96.3) RU/ml, with 11.5% of cases above 130 RU/ml.

### Clinical Events

Mean follow-up was 2.15±0.99 years. Seventy-seven patients developed the outcome. Of these, 12 suffered two events and 5 developed 3 events. There were 39 NSTEACS, 4 STEMI, 8 strokes, 10 transient ischemic attacks, 16 episodes of heart failure and 22 deaths. Eight deaths were due to cardiovascular causes, four to malignancies and one to infection, renal failure, bowel ischemia, gastrointestinal bleeding and pancreatitis. Five deaths were of unknown etiology.

### Relationship of Calcidiol and FGF-23 Plasma Levels with Prognosis in the Whole Population

**Figure 1:**
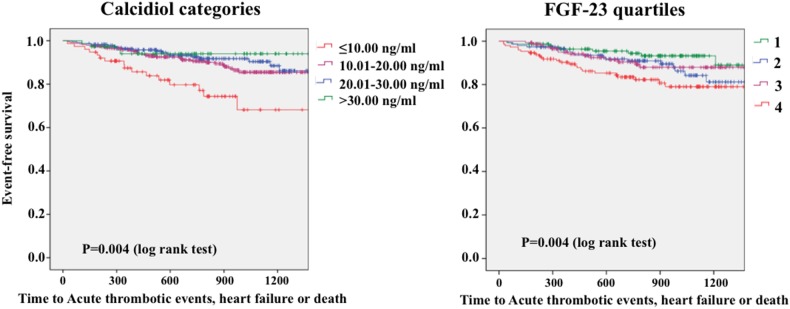
Kaplan-Meier survival curves showing the differences in outcome in the whole population. Low calcidiol (left) and high FGF-23 (right) plasma levels are associated with a worse prognosis.

Kaplan-Meier curves showed that prognosis was worse as plasma calcidiol levels decreased (p = 0.004) (Figure 1, left). Conversely, progressively increasing levels of FGF-23 were associated with poorer prognosis (p = 0.004) (Figure 1, right). Patients that developed the outcome had higher FGF-23 and parathormone plasma levels but low calcidiol levels and glomerular filtration rate than those who remained stable (Table 1). Phosphate, high sensitivity C-reactive protein and lipid levels showed no differences between both groups. In multivariate analyses, FGF-23 plasma levels and calcidiol categories remained as independent predictors of outcome, along with age and hypertension (Table 2).

**Table 2:** Multivariable Cox model for the incidence of the outcome in the whole population.

	Hazard Ratio	95% CI		P value
		Lower	Upper	
**Age**	1.05	1.03	1.07	<0.001
**Hypertension**	2.61	1.22	5.58	0.013
**FGF-23**	1.13*	1.04*	1.23*	0.005
**Calcidiol**	0.67	0.48	0.94	0.021

**CI:** Confidence Interval; **FGF-23:** Fibroblast Growth Factor-23.

*Increase in hazard ratio per 100 RU/ml.

Calcidiol is assessed as categorical variable. Reference category is: 0.00–10.00 ng/ml.

### Influence of the Relationship between Calcidiol and FGF-23 Levels on Prognosis

**Figure 2:**
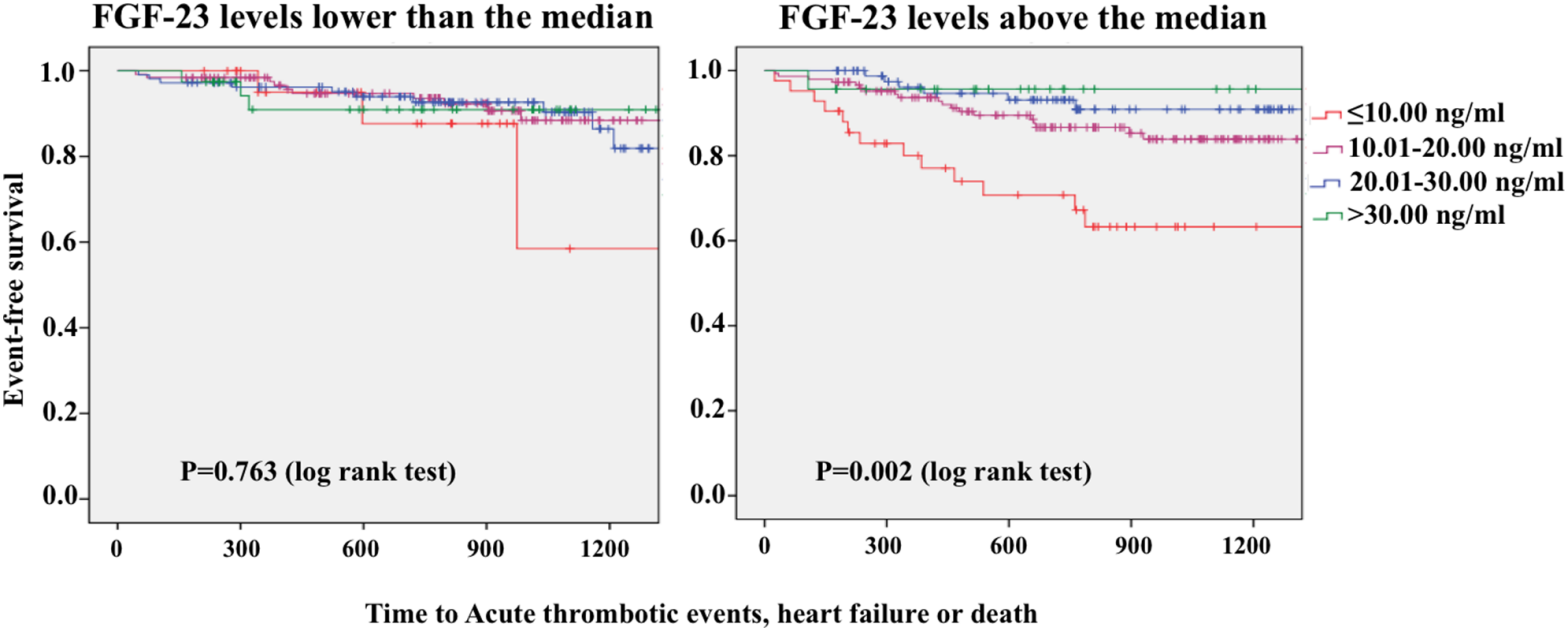
Kaplan-Meier survival curves showing the differences in outcome as predicted by calcidiol in population subgroups according to FGF-23 plasma levels. In patients with FGF-23 lower than or equal to the median (69.9 RU/mL), calcidiol plasma levels are no longer associated with prognosis (left). However, calcidiol levels are still strongly associated with a worse prognosis in the subgroup with FGF-23 levels above the median (right).

To explore this relationship, we divided the population in two groups according to median FGF-23 levels (69.9 RU/mL). Kaplan-Meier curves showed that the relationship between the different calcidiol categories and outcome observed in the whole population did not hold in patients with FGF-23 levels equal or below the median (p = 0.763) (Figure 2, left). In contrast, this association became more evident in the subgroup with FGF-23 levels above the median (p = 0.002) (Figure 2, right).

When Cox multivariate analysis was repeated in patients with FGF-23 plasma levels above the median, calcidiol categories were still independent predictors of the outcome (Table 3). However, calcidiol was no longer an independent predictor of the outcome in patients with FGF-23 plasma levels below the median (Table 4).

**Table 3:** Multivariable Cox model for the incidence of the outcome in patients with FGF-23 plasma levels higher than the median.

	Hazard Ratio	95% CI		P value
		Lower	Upper	
**Age**	1.06	1.03	1.09	<0.001
**Hypertension**	3.60	1.10	11.78	0.011
**Calcidiol**	0.50	0.31	0.80	0.003

**CI:** Confidence Interval.

Calcidiol is assessed as categorical variable. Reference category is: 0.00–10.00 ng/ml.

**Table 4:** Multivariable Cox model for the incidence of the outcome in patients with FGF-23 plasma levels equal or lower than the median.

	Hazard Ratio	95% CI		P value
		Lower	Upper	
**Age**	1.04	1.01	1.07	0.023
**Hypertension**	2.54	0.92	6.98	0.052
**Calcidiol**	1.03	0.62	1.71	0.904

**CI:** Confidence Interval.

Calcidiol is assessed as categorical variable. Reference category is: 0.00–10.00 ng/ml.

### Relationship between Mineral Metabolism Parameters and Estimated Glomerular Filtration Rate, Age, Sex and C-reactive Protein

There was a modest inverse correlation between estimated glomerular filtration rate and both FGF-23 (r = −0.351; p<0.001) and parathormone (r = −0.255; p<0.001). Calcidiol (r = 0.000; p = 1.000) and phosphate (r = −0.034; p = 0.376) were not correlated with renal function. Similarly, age showed a mild correlation with FGF-23 (r = 0.209; p<0.001) and parathormone (r = 0.236; p<0.001), but it did not correlate with calcidiol (r = −0.036; p = 0.343) and phosphate levels (r = −0.031; p = 0.420).

Compared to men, women showed lower levels of calcidiol (17.1 [12.3–23.3] versus 19.3 [14.1–24.9] ng/ml; p = 0.003) but higher FGF-23 (84.6 [65.6–124.2] versus 66.8 [52.5–89.6)] RU/mL; p< 0.001), parathormone (70.4 [52.1–91.2] pg/mL versus 56.9 [43.5–73.8]; p<0.001) and phosphate (3.4 [3.1–3.8] versus 3.1 [2.8–3.5] mg/dl; p<0.001).

C-reactive protein showed a mild correlation with FGF-23 (r = 0.159; p<0.001) and calcidiol (r = −0.111; p = 0.003), but not with parathormone (r = −0.009; p = 0.814) or phosphate (r = 0.050; p = 0.191).

By multivariate linear regression analysis we investigated how much of calcidiol (Table 5) and FGF-23 plasma levels (Table 6) were explained by age, sex, glomerular filtration rate, and parathormone, phosphate, and high sensitivity C-reactive protein plasma levels. The R^2^ value was 0.121 for calcidiol and 0.187 for FGF-23 plasma levels, indicating that the variables studied explained only a low percentage of the variation of these parameters.

**Table 5:** Multivariate linear regression analysis assessing the relationship of calcidiol with other variables studied.

	b coefficient	95% CI		P value
		Lower	Upper	
**Intercept**	54.69	47.26	62.11	<0.001
**Log PTH**	−7.47	−8.99	−5.96	<0.001
**GFR**	−0.06	−0.09	−0.02	0.001
**Log hs-CRP**	−0.67	−1.16	−0.18	0.007

R^2^ = 0.121.

Age, sex, Glomerular filtration rate **(GFR)**, Log parathormone **(PTH)**, Phosphate and Log high sensitivity C-reactive protein **(hs-CRP)** were assessed in the model.

**Table 6:** Multivariate linear regression analysis assessing the relationship of FGF-23 with other variables studied.

	b coefficient	95% CI		P value
		Lower	Upper	
**Intercept**	3.87	3.18	4.56	<0.001
**Sex**	−0.15	−0.26	−0.04	0.007
**Log PTH**	0.20	0.08	0.32	0.001
**Phosphate**	0.12	0.04	0.20	0.005
**GFR**	−0.01	−0.01	−0.01	<0.001
**Log hs-CRP**	0.06	0.03	0.10	0.001

R^2^ = 0.187.

Age, sex, Glomerular filtration rate **(GFR)**, Log parathormone **(PTH)**, Phosphate and Log high sensitivity C-reactive protein **(hs-CRP)** were assessed in the model.

## Discussion

This study demonstrates for the first time that the combination of calcidiol and FGF-23 plasma levels is a strong predictor of adverse events in patients with CAD.

Our report also shows that the abnormalities of mineral metabolism are present in a remarkable number of patients with CAD, even although only one-fifth of them had an estimated glomerular filtration rate lower than 60 mL/min/1.73 m^2^. The presence of these alterations is not merely a marker of decreased renal function, as the estimated glomerular filtration rate did not correlate with calcidiol and phosphate and showed only a mild correlation with FGF-23 and parathormone.

We have also demonstrated that low vitamin D levels were independently associated with an adverse prognosis in patients with CAD. These findings are in accordance with several previous studies. Three meta-analyses, including patients with and without cardiovascular disease at baseline, found an inverse association between circulating 25-hydroxyvitamin D levels and risk of cardiovascular disease and cardiovascular death [11]–[13]. Also, low plasma vitamin levels have been associated with the severity of CAD [14], [15]. On the other hand, high vitamin D levels are associated with decreased risk of CAD in the general population [6], [16]. In fact, low vitamin D may lead to endothelial dysfunction, inflammation, activation of the renin-angiotensin system, vascular smooth muscle cell proliferation and vascular calcification and myocardial infarction [5], [14], [17]–[19]. Very recently, Siasos et al found that vitamin D deficiency was an independent predictor of adverse outcome in patients with CAD undergoing coronary angiography [20]. However, the sample size was lower than that of the present study, and the other components of mineral metabolism were not assessed.

Conversely, vitamin D plasma levels failed to predict the incidence of cardiovascular events and mortality in patients with stable CAD randomized to two doses of atorvastatin [21]. It was hypothesized that statins could overcome the vascular damage related to vitamin D deficiency. However, in our study, most patients were receiving statins and, in spite of this, calcidiol levels predicted an adverse outcome.

Calcidiol levels showed a mild correlation with C-reactive protein and no correlation with estimated glomerular filtration rate, suggesting that calcidiol levels provide prognostic information that is unrelated to these factors. Nevertheless, in our patients, none of these variables were independent predictors of an adverse prognosis. Indeed, C-reactive protein levels were not different between both groups at baseline. Although this molecule has been suggested to be an independent risk predictor, not all the studies confirm this idea [22]. Regarding glomerular filtration rate, although patients developing events displayed lower values of this parameter at baseline, it was also not an independent predictor of adverse prognosis at multivariate analysis. It seems possible that calcidiol and also FGF-23 levels are more direct markers of the abnormalities leading to cardiovascular damage in renal dysfunction than glomerular filtration rate. Of interest, lipid levels were not different between patients that met the primary outcome and those who remained stable. Although the prognostic implication of this parameter is well known, it is possible that this information is less useful in patients on secondary prevention as, in this setting, intensive statin therapy is advised, and lipid levels become more homogeneous and thus may have less ability to discriminate prognosis.

A second finding of this study was that high FGF-23 plasma levels were also a strong independent predictor of adverse prognosis. Plasma levels of this phosphaturic hormone gradually rise in chronic kidney disease to prevent hyperphosphatemia. FGF-23 is also a potent inhibitor of parathormone secretion and reduces vitamin D levels trough decreased production and enhanced catabolism [2], [23]. High FGF-23 levels are associated with an enhanced risk of mortality in patients undergoing hemodialysis [24]. Although these levels could reflect the risk associated with vitamin D deficiency and hyperparathyroidism, it has been hypothesized that this factor itself could contribute to cardiovascular disease. In this sense, FGF-23 has been related with endothelial dysfunction, increased arterial stiffness, high atherosclerosis burden in the community, vascular calcification, left ventricular hypertrophy, and progression of renal disease [3], [25]–[29].

Several studies have reported different results about the relationship between FGF-23 and CAD. In the Uppsala Longitudinal Study of Adult Men (ULSAM), high FGF-23 blood levels were also associated with increased risk of cardiovascular mortality in the community, even after adjustment for glomerular filtration rate [28]. On the other hand, no relationship between cardiovascular risk and FGF-23, parathormone, and phosphate plasma levels was found in men without cardiovascular disease at baseline after adjusting for 25-hydroxyvitamin D levels and clinical data in the Health Professionals Follow-up Study (HPFS) [30]. However, the profile of these populations was quite different. In the ULSAM study, 27% of cases had a previous diagnosis of cardiovascular disease, glomerular filtration rate was lower, and age and FGF-23 plasma levels were higher than in the HPFS study. Then, it seems that FGF-23 may be specially effective as a prognostic marker in populations at high risk of adverse outcomes. In this regard, our study included population at high risk, with a previous diagnosis of CAD and a glomerular filtration rate more similar to that of patients from the ULSAM study. Also, our patients had lower vitamin D and high FGF-23, PTH and phosphate plasma levels than those of the HPFS study. Finally, the Heart and Soul Study showed a relationship between FGF-23 and cardiovascular risk patients with CAD and average renal function [4]. The present study extends this observation investigating also the relationship between the prognostic value of FGF-23 and vitamin D plasma levels.

The most relevant finding of this study was that the ability of low calcidiol levels to predict an adverse outcome was limited to those cases where FGF-23 was higher than the median, with this predictive power disappearing when FGF-23 was equal or below these levels. Of interest, the prognostic information provided by both metabolites was independent as evidenced by the multivariate analysis. This finding is important, since a wide collection of clinical and analytical data have been assessed, including high sensitivity C-reactive protein and lipid levels.

As explained, FGF-23 contributes to decrease vitamin D levels. A recent work has shown that predialysis patients with high FGF-23 and low vitamin D levels have higher probability of doubling serum creatinine or initiating dialysis [31]. Similarly, a study carried out in patients with advanced chronic kidney disease showed that low plasma vitamin D levels were associated with death and initiation of long-term dialysis, but FGF-23 levels attenuated this relationship [32]. However, this is the first time that this association has been found related to an adverse clinical outcome in patients with CAD. This relationship may be important in understanding why some studies have found that low vitamin D levels are not associated with increased cardiovascular risk [21]. It may also be relevant to explain the controversial results obtained in studies with vitamin D supplementation [33]–[35] and it should be taken into account in the design of future trials, as the effect of vitamin D could depend on FGF-23 plasma levels. Furthermore, as vitamin D supplementation may increase FGF-23 levels [36], monitoring this factor could be of interest in future clinical trials. Finally, both calcidiol and FGF-23 plasma levels could be considered in the design of future risk scales for patients with CAD.

There are limitations in this study. 1) Urinary albumin was not measured in our population, so earlier stages of chronic renal disease could have gone undetected. 2) Excluding patients not clinically stable during the first days after the index event could introduce a certain bias, because these patients would probably have a worse prognosis. Nevertheless, only nine percent of cases were excluded for this reason.

## Conclusions

Low calcidiol plasma levels are an independent predictor of adverse outcome in patients with stable CAD who have high FGF-23 levels. Assessment of both, calcidiol and FGF-23 plasma concentration could be of interest in risk prediction in these patients.
